# Impact of a silver filter with deep learning reconstruction on non-contrast MDCT of adrenal glands in terms of image quality, density values and radiation dose

**DOI:** 10.1186/s12880-026-02598-4

**Published:** 2026-07-22

**Authors:** Catherine Roy, Quentin le Duff, Luc Mertz, Hugo Jaillet, Aissam Labani, Sébastien Moliére, Mickael Ohana

**Affiliations:** 1 Department of Radiology B, University Hospital of Strasbourg – New Civil Hospital, 1, place de l’ hôpital BP 426, Strasbourg Cedex, 67091 France; 2 Medical Physics Department, University Hospital of Strasbourg – New Civil Hospital, 1, place de l’ hôpital BP 426, Strasbourg Cedex, 67091 France

**Keywords:** Deep learning reconstruction, X-rays tube filtration, Non injected abdominopelvic MDCT, Radiation dose

## Abstract

**Background:**

To investigate whether the use of a silver beam filtration with deep learning reconstruction (DLR) impact the normal adrenal gland on unenhanced MDCT in terms of density, image quality and radiation dose.

**Methods:**

This unicentric retrospective study evaluated two unenhanced MDCT acquisitions of 123 patients performed with a conventional filtration (Group A) and with a silver filtration (Group B) on a 320-detector row CT scanner with DLR. A total of 246 series, corresponding to 492 adrenal glands, were analyzed. We recorded the HU values of the normal adrenal gland, subcutaneous fat and extra corporeal air as well as the radiation dose and assessed image quality.

**Results:**

The subjective image quality was excellent, with no significant difference between the two acquisitions with a mean score on the Likert scale of 4.6 ± 0.3 and 4.1 ± 0.6 for groups A and B, respectively (*p* = 0.09). Interobserver agreement for image quality was good and quite similar for both groups (group B: k= 0.86; group A: k= 0.90). The mean density of adrenal gland was 15.65 ± 5.5 HU with conventional filtration (Group A) and 13.05 ± 4.5 HU for silver filtration (Group B). For the right adrenal gland, the median values were 15.8 HU and 12.9 HU for group A and B, respectively (p=0.01). For the left adrenal gland, the median values were 15.5 HU and 13.2 HU for group A and B, respectively (p=0.03). A subgroup of 135 adrenal glands (including both right and left side) with a density value ≤ 15HU using conventional filtration (Subgroup A) showed a statistically significant lower median value with silver filtration (Subgroup B). Inside this ≤ 15 HU subgroup, 59 adrenal glands had a density value equal or lower than 10 HU of which 48 belonged to subgroup B. A reduction in HU values of 18% was observed with the silver filter in the overall cohort (492 adrenal glands), and 26% in the subgroup ≤ 15 HU. Similarly, the mean score for image noise was 7.6 ± 1.5 for conventional filter and 8.3 ± 0.9 for silver filtration, with no statistically significant difference (*p* = 0.08). Radiation dose was significantly reduced by 35% in examinations performed with silver filtration when compared to conventional filter.

**Conclusion:**

The spontaneous density of normal adrenal gland is lower than previously reported. Implementing silver filtration with DLR in CT acquisition achieves a significant radiation dose reduction while preserving image quality, along with a decrease in adrenal gland HU values, often below 10 HU. This must be taken into account as this is the threshold of the key reference for adenoma.

**Trial registration:**

This study is part of a routine clinical practice. This unicentric retrospective study was approved by our local institutional review board, and written informed patient consent was not required. The patients were orally informed that their second CT examination will be performed using the newest technology available on the CT machine in our department.

## Background

Attempts to reduce radiation dose is a constant purpose for all CT manufacturers [1–3]. In the past, iterative reconstructed (IR) methods using copper filter have been developed to reduce the dose; however, low-dose CT images remained significantly, with poor spatial resolution and a plastic-like appearance [4, 5].

More recently, deep learning reconstruction (DLR) algorithms have been introduced, enabling a significant reduction of the radiation dose while maintaining an excellent image quality and offering a faster reconstruction speed compared with previous reconstruction types [6–8]. Few studies have demonstrated the positive impact of silver filter in combination with deep learning reconstruction on lung parenchyma in terms of image quality and radiation dose reduction [9, 10]. A recent study [11] reported a significant reduction of the HU values with silver beam filtration and DLR in some abdominal organs, compared with standard filtration with IR on contrast-enhanced CT examinations.

Current radiological references do not provide a precise HU range for normal adrenal gland tissue on unenhanced CT, except in one ancient paper published during the previous century [12]. Despite the fact that the noncontrast value of normal gland is not routinely measured or reported as a clinical parameter; the key reference point for adrenal adenoma is a HU equal or lower than 10 on non-contrast CT.

The aim of this study was to investigate in a large cohort of patients, the density of normal adrenal glands and to determine whether the use of the silver beam filtration combined with deep learning reconstruction impacts the unenhanced adrenal gland in terms of density, image quality and radiation dose.

## Methods

**Fig. 1:**
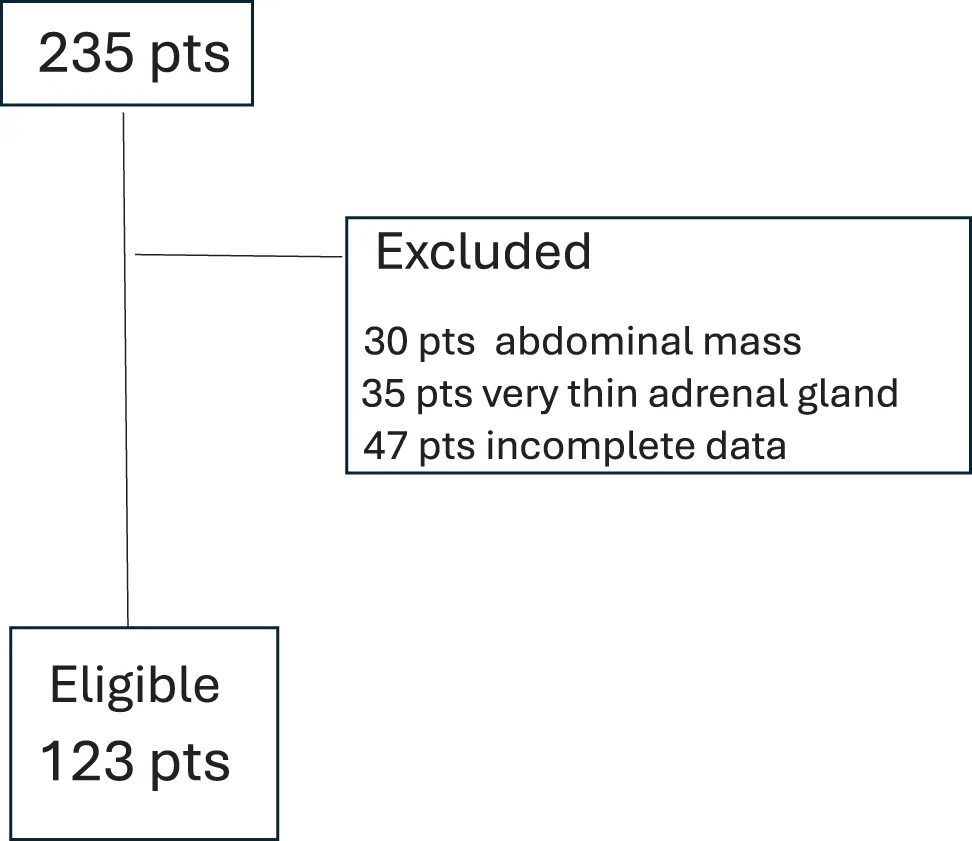
Flow chart of patients with final cohort

From January 2025 to January 2026, among a large group of 235 patients explored in the context of abdominal pain, search for tumoral lesion, diabetes or renal medical disease, we excluded 112 patients (30 patients with an abdominal mass whatever was the organ or the diagnosis, 35 patients with a very thin adrenal glands unreliable to obtain UH value and 47 patients previously explored in another radiological center). All patients had a Body Mass Index (BMI) between 18.5 kg/m2 and 25 kg/m2. Finally, 123 patients that means 246 series (492 adrenal glands) were recorded for our study with previous examination performed in our department. The flow chart is shown in Fig. 1.

This unicentric retrospective study was approved by our institutional review board and written informed patient consent was not required. The patients were orally informed that their second CT examination will be performed using the newest technology available on the CT machine in our department.

We evaluated the unenhanced MDCT abdominal acquisition, which was the initial part of our CT protocol for the clinical context of the same patient. The time interval between the two examinations was between 9 and 3 months. Both examinations were realized on a 320-detector row CT scanner (Aquilion One Insight Edition Version 1.7 (Canon Medical Systems, Otawara, Japan).

The unenhanced acquisition of the first CT examination (Group A) was performed with the conventional Aluminum/Copper filtration. The unenhanced acquisition of the second CT examination (Group B) was performed with the new Aluminum/Silver filtration. The acquisition started at the top of the diaphragm and ended under the pubic symphysis. Helical scan parameters were kept identical between the two examinations for each patient: fixed 120 kV, automatic exposure control (SURE Exposure 3D Canon Medical) based on the scout image attenuation, with a mA range from 80 to 650 mA and a reconstructed images using a soft tissue kernel with deep learning reconstruction (DLR) technique named Advanced intelligent Clear-IQ Engine (AiCE). The noise index was set at « standard −1 » that is 15.0 for SD in Group B according to the manufacturer’s setting for silver filter and at « standard » that is 11.5 for SD in Group A according our routine clinical practice with conventional filter. The acquisition time varied from 7 s to 9 s (Table 1).

**Table 1:** MDCT acquisition and reconstruction parameters for both groups. Al: Auminium; Cu: copper; Ag: silver. DLR: deep learning reconstruction; AIDR 3D: adaptive iterative dose reduction 3-dimensional; AiCE: advanced intelligent clear-IQ engine; kVp: kilovoltage peak; mA: milliampere. SD: standard deviation

Parameters	Group A (AiCE) DLR	Group B (AiCE) DLR
Acquisition mode	Helical	Helical
Tube voltage (kVp)	120	120
Tube current (mA)	80–650	140–550
Beam filtration	**Al/Cu**	**Al/Ag**
Modulation SD	11.5 (standard)	15 (standard −1)
Collimation (mm)	0.5 × 80 row	0.5 × 80 row
Rotation time (s)	0.5	0.5
Field of view (mm)	380 × 500	380 × 500
Slice thickness (mm)	1.0/0.8	1.0/0.8
Interval (mm)	0.8	0.8
Pitch	0.813	0.813
Matrix size	512 × 512	512 × 512

Quantitative analysis

All images were analyzed on the VITREA workstation version 7.11 (Canon Medical Informatics, Minneapolis, Minnesota, USA)

Abdominal CT images were assessed using soft-tissue window settings (window level, 40 HU; window width, 400 HU).

**Fig. 2:**
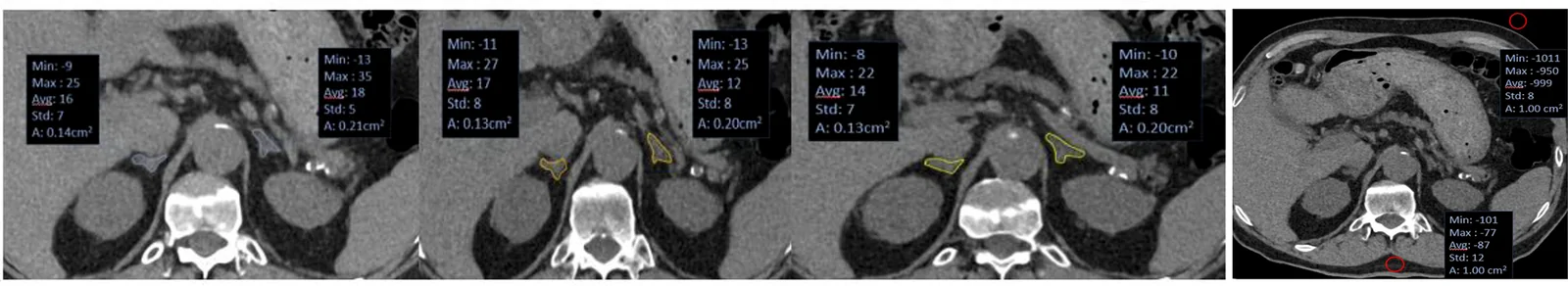
Example of recording the average HU value with a manual drawing delineating the adrenal gland from 3 contiguous slices and circular ROI inside fat and extracorporeal air. MDCT acquisition with silver beam filtration

The measurements were conducted on the reconstructed 1 mm axial images by a radiologist with 4 years of experience (x). A manual drawing of ROI encompassing thoroughly the adrenal gland was performed on both sides. The average CT density in Hounsfield units (HU), along with the standard deviation of the CT value (SD), was obtained from three measurements from 3 contiguous slices (Fig. 2).

A circular ROI of 1.00 cm2 was placed in the subcutaneous fat behind the erector spinae muscle and inside the extra corporeal air in front of the anterior abdominal wall in the same location for both examinations.

Noise was defined as the mean of the standard deviation (N) of the signal measurement in extracorporeal air.

The signal-to-noise ratio (SNR) and contrast-to-noise ratio (CNR) were calculated using the following formulas using the mean value extracted from the three ROIs:

SNR = mean attenuation of the interested organ/standard deviation in extra-corporal air (N)

CNR = (mean attenuation of the interested organ–mean attenuation of the subcutaneous fatty tissue)/standard deviation of the subcutaneous fatty tissue

The dose-length product (DLP) values and Volume Computed Tomography Dose Index (CTDIvol) displayed on the CT console were recorded.

Qualitative analysis

Image quality was assessed by two radiologists with 4 (x) and 25 years (xx) of experience in abdominal imaging and knowledge on CT images with DLR. They were blinded to the patient data and acquisition parameters.

Readers were asked to subjectively and independently rate the overall image quality on the two sets of MDCT examinations using a five-point Likert scale ranging from 1 to 5: 1 = very dissatisfied image quality, 2 = dissatisfied image quality, 3 = moderate image quality, 4 = satisfied image quality, 5 = very satisfied image quality [13].

Disagreements were resolved by consensus with a third reader (xxx).

Statistical analysis

Statistical analysis was conducted using the XLSTAT statistical software (Addinsoft, Microsoft Excel, version 2019). Interobserver agreement for the qualitative analysis of image quality was assessed using quadratic Cohen’s weighted kappa statistics (k). Kappa statistics were calculated using 95% confidence intervals. Hereby, kappa values < 0 were considered as indicating no agreement, 0.01–0.20 as poor, 0.21–0.40 as fair, 0.41–0.60 as moderate, 0.61–0.80 as substantial, and 0.81–1.00 as an excellent agreement.

The student’s paired t-test was used to evaluate differences between the two examinations in terms of HU values, SNR, CNR, DLP, and CTDIvol. The paired t-tests were performed separately for the right and left glands to reflect anatomical reality. Statistical difference was considered as significant at *p* ≤ 0.05.

## Results

Patient population

The final cohort (Fig. 1) of 123 patients, enrolled in this study that means 246 series (492 adrenal glands), was composed by 56 women and 67 men with a mean age of 56 ± 24.5 years old. There was no change in the BMI of the patients between the two examinations.

Image quality

The subjective image quality was judged excellent without significant difference between both acquisitions with a mean score on the Likert scale of 4.6 ± 0.3 and 4.1 ± 0.6 for group A and B, respectively (*p* = 0.09).

No artifacts were present on both groups.

The interobserver agreement on image quality was excellent and quite similar for both groups (group B: k = 0.86; group A: k = 0.90).

Image analysis

The size of all adrenal glands was within normal limits on our visual radiological analysis. For all cases, it was found a lower HU values for silver filter than for conventional filter with a statistically significant difference.

The mean density of the whole series of adrenal gland was 15.65 ± 5.5 HU with conventional filtration (Group A) and 13.05 ± 4.5 HU for silver filtration (Group B).

For the right adrenal gland, it was found a median value of 15.8 HU and 12.9 HU for group A and B, respectively (*p* = 0.01)For the right adrenal gland, the median values were 15.8 HU and 12.9 HU for group A and B, respectively (*p* = 0.01). (Table 2).

**Table 2:** Median HU value ± standard deviation with SNR and CNR of the overall data and a subgroup ≤ 15 HU. Groups A and B: whole data of 123 patients (246 series, 492 adrenal glands); subgroups A and B (≤15HU): data of 68 patients (136 series, 135 adrenal glands)

	Group A Al/Cu filter HU value	Group B Al/Ag filter HU value	p value
Right adrenal gland	15.8 (95% CI: 10.3–21.3)	12.9 (95% CI:7.0–18.8)	0.01
Left adrenal gland	15.5 (95% CI:10.0–21.0)	13.2 (95% CI: 7.3–19.1)	0.03
Subcutaneous fatty tissue	−94.0 (95% CI: −103.5– − 91.4)	−85.2 (95% CI: −83.5– −87.5)	0.0003
	Subgroup A (≤15HU) **Al/Cu** filter HU value	Subgroup B (≤15HU) **Al/Ag** filter HU value	
Right adrenal gland	9.2 (95% CI: 3.7–14.7)	6.9 (95% CI: 1.0–12.8)	0.003
Left adrenal gland	8.7 (95% CI: 3.2–14.2)	6.8 (95% CI: 1.9–11.7)	0.02
**SNR**	7.14 (95% CI: 6.29–7.22)	6.58 (95% CI: 5.68–7.04)	0.004
**CNR**	10.7 (95% CI: 9.2–12.3)	10.2 (95% CI: 9.2–11.3)	0.09

For the left adrenal gland, it was found a median value of 15.5 HU and 13.2 HU for group A and B, respectively (*p* = 0.03). For the left adrenal gland, the median values were 15.5 HU and 13.2 HU for group A and B, respectively (*p* = 0.03).

There was no significant difference in HU values between the right and left glands, regardless of the group.

A subgroup of 135 adrenal glands including the right and left side with a density value ≤ 15HU with conventional filtration (subgroup A) had a statistically significant lower median value with silver filtration (subgroup B). Inside this subgroup ≤ 15 HU, 59 adrenal glands had a density value equal or lower than 10 HU of which 48 are in the subgroup B.

We have found a lower HU values with the silver filter of 18% considering the entire cohort of 492 adrenal glands, and of 26% with the subgroup ≤ 15 HU (135 adrenal glands).

Our findings indicated that the SNR was slightly higher in group A, with a statistically significant difference. However, for the CNR no statistical difference was found (Table 2), even if the CNR result was slightly higher for the group A.

Similarly, the mean score for image noise was 7.6 ± 1.5 for Al/Cu (Group A) and 8.3 ± 0.9 for Al/Ag (Group B) with a percentage decrease of 0.08% and no statistically significant difference (*p* = 0.08)

Radiation dose

**Fig. 3:**
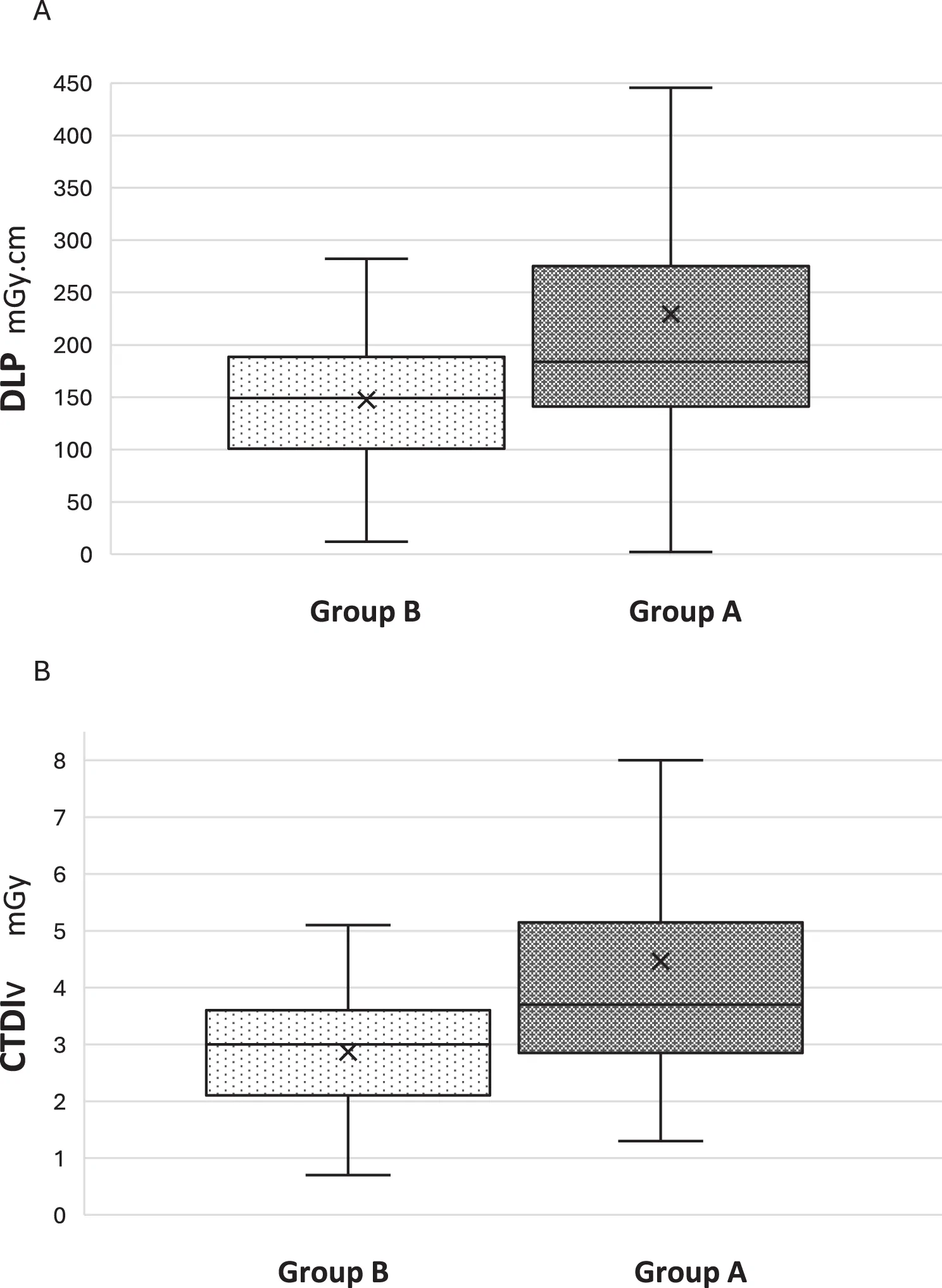
Radiation dose DLP and CDTIvol recorded between Al/Cu (group A) and Al/Ag (group B). Box-and-whisker diagrams show mean values ± standard deviation and minimum and maximum values. (**A**): DLP, (**B**): CTDIvol

The mean DLP and CTDI_vol_ were 229.28 ± 37.5 mGy.cm and 4.46 ± 1.02 mGy in the control group A (Al/Cu filter -Group A); 147.28 ± 21.8 mGy.cm and 2.86 ± 0.53 mGy in the group B (Al/Ag filter- Group B), respectively (*p* < 0.001) (Fig. 3). The radiation dose reduction is higher than the increase in noise.

The total applied radiation dose in CTDI_vol_ was found to be significantly lower by 35% with silver filter when compared to the conventional filter.

## Discussion

The filtration of the X- ray beam released from the tube is essential to modulate the energy spectrum penetrating the body, allowing an acceptable compromise between the image quality and the radiation dose delivered.

The main action of a filter is to reduce unnecessary skin exposure by the eliminating low-energy photons and producing a more uniform beam.

The commonly used bowtie filter is composed by the couple aluminum and copper which creates an X-ray spectrum with a relative wide range of energies.

Silver has been recently proposed for filtering the X-rays in CT. Its physical properties with a higher K-edge of 25.5 keV and high atomic number (Z = 47) harden the X-rays spectrum more than the copper (K-edge of 8.9 keV, Z = 29). This new type of CT bowtie filter is composed by the usual aluminum contiguous with a silver layer. It eliminates the majority of the low-energy photons from a polychromatic X-ray beam which do not contribute to the image quality but increase dose and scatter. As a result, there is a shift toward a narrower beam with a higher and more uniform energy level. Theoretically, the consequence is a better image quality by reducing the noise, a higher SNR and a reduction of the radiation dose received by the patient.

A positive impact has recently been reported for lung parenchyma screening [9, 10]. In combination with the DLR reconstruction, there is an outstanding additional improvement in image quality, an excellent analysis of structures with low contrast and a stronger reduction of the noise. It has been demonstrating to be clearly superior, providing sharper images compared with conventional filtration using the foregoing hybrid iterative reconstruction (HIR) approach.

Based on chest phantoms, studies have demonstrated a high radiation dose reduction from 30% to 75% depending on the reference used for reconstruction, while maintaining image quality [14–16].

The clinical study of Otgonbaatar [11] performed on contrast-enhanced abdominopelvic CT reported a significant reduction of the effective dose of 59% with silver filter with DLR compared with standard filter and IR. However, unlike our study, they have correlated different patients with similar BMI and the examinations were performed on different manufacturers and with different acquisition parameters.

Our results indicate an interesting reduction in radiation dose with silver filtration, but less impressive than in the study of Golbus [9] on chest, where it was found a considerable reduction in radiation evaluated at 85.5%. However, in that paper, the correlation had been done with a standard acquisition with a hybrid iterative reconstruction (HIR) whereas in our study, DLR was used for both examinations. In addition, their cohort was composed by patients with thin « biotype » but our patients were « healthy weighted » meaning a higher BMI.

It is commonly admitted that the normal adrenal gland has a uniform HU value of soft tissue, similar to that of the liver and spleen on unenhanced CT, estimated from 25 to 40 UH [17]. However, current radiology references do not provide a precise HU range of value for normal adrenal gland on unenhanced CT. Only one ancient paper published in 1997, studying adrenal masses has used normal gland as reference. This paper reported a mean attenuation of the normal adrenal gland on unenhanced CT scans of 24 HU ±3, based on a small cohort of 40 patients [12]. On the other hand, the key reference point to diagnose a lipid-rich adrenal adenoma is an attenuation value ≤ 10 HU on unenhanced CT [18].

To the best of our knowledge, our work is the first study to provide a precise density of normal adrenal gland on unenhanced MDCT based on a large cohort, using a recent imager with and without silver beam in the same patient.

Interestingly, we found a mean density clearly inferior to that suggested in literature with conventional filtration when using silver beam filtration, showing a significant difference of 18%. In the selected subgroup ≤ 15HU, we have recorded many HU values ≤ 10HU more numerous with the silver filtration. The differences between our results and previously reported thresholds may be explained by technological advances over the past three decades.

We can suggest that this value may serve as a reference for the normal adrenal gland. Consequently, a value ≤ 10 HU in a gland of normal size should be considered consistent with normal tissue. However, this interpretation should be made with caution in cases of collision tumors or glands at the limit of normal size.

Our subjectively evaluated image quality was excellent for both groups. There was no consequence of the higher threshold of the modulation chosen for silver beam that reflects the slightly higher SD of the HU values observed in group B. The explanation could be the association of the noise reduction and the DLR technique. As the noise setting index differs between the two groups, reflecting our clinical practice for group A, only a small proportion of the radiation dose reduction can be attributed to it according to our data. We believe that the most important part of the radiation reduction is due to the silver filter.

In agreement with lung studies, there was no plastic or « artificial » appearance of images which had been observed in the past with strong iterative reconstruction techniques.

The shift toward a higher energy level of the X rays beam could be proposed as a potential explanation to explain the reduction in HU with silver filtration. Some previous publications [19, 20] performed on phantoms containing different tissue equivalent inserts have demonstrated that changes in kVp directly impacted the HU measured. Any increase in the kVp results in a corresponding decrease in HU. A slight reduction in CT numbers has been observed at a higher kVp values, particularly for tissues with HU values below 60, which represents the density of most human soft tissues. The highest reduction was observed for the lowest positive density, such as water/urine.

As a remark, the use of silver beam filtration is not suitable for injected exams because at a higher energy level the intensity of the iodine uptake is significantly reduced, leading to misdiagnosis. This statement is noticed in the study ofOtgonbaatar [11] performed on injected abdominopelvic examinations.

Concerning the SNR, our results showed a statistically significant lower SNR with silver filtration compared to the group A. This is in agreement with others studies [9–11]. However, the difference is not large enough to be clinically significant.

Our study has some limitations.

Firstly, this retrospective study was conducted in a single institution and focused on the radiation dose and image quality on non-contrast MDCT without including pathological cases. Moreover, the gold standard for diagnosing adrenal adenoma is based on unenhanced CT. However, we demonstrated that a value ≤ 10UH can be found in normal-size**d** gland**s** more frequently when using a silver filtration

Secondly, our scan parameters were identical except for the modulation, but our choice was to keep the parameters defined by the CT manufacturer for the silver beam However, this had no impact on the image quality.

## Conclusions

To the best of our knowledge, this is the unique recent study performed on a large cohort that provides reference HU values for the normal adrenal glands. Implementing the silver filtration with DLR achieves a significant radiation dose reduction while preserving image quality along with a decrease in HU values in normal gland with a certain percentage falling below 10 HU. These findings should be taken into account in clinical routine.

## Data Availability

Data required for this study may be made available by the author(s) upon reasonable request.

## Abbreviations

AiCE: Advanced intelligent clearIQ engine
AIDR3D: Adaptive iterative dose reduction
DLR: Deep learning reconstruction
SNR: Signaltonoise ratio
CNR: Contrast- to- noise ratio
Al: Aluminum
Cu: Copper
Ag: Silver
Al/Ag: Silver filter
Al/Cu: Copper filter
IR: Iterative reconstruction
HIR: Hybrid iterative reconstruction
CTDIvol: Volume computed tomography dose index
BMI: Body mass index
SD: Standard deviation
ROI: Region of interest

## References

[1] Smith-Bindman R, et al. Radiation dose associated with common computed tomography examinations and the associated lifetime attributable risk of cancer. Arch Intern Med. 2009;169(22):2078–86. 10.1001/archinternmed.2009.427. PMC4635397 20008690

[2] Shubayr N, Alashban Y. Estimation of radiation doses and lifetime attributable risk of radiation-induced cancer in the uterus and prostate from abdomen pelvis CT examinations. Front Public Health. 2023;10:1094328. 10.3389/fpubh.2022.1094328. eCollection 2022. PMC9868812 36699908

[3] Mathews JD, Forsythe AV, Brady Z, Butler MW, Goergen SK, Byrnes GB, et al. Cancer risk in 680 000 people exposed to computed tomography scans in childhood or adolescence: data linkage study of 11 million Australians. BMJ. 21 May 2013;346(1):f2360. 10.1136/bmj.f2360. PMC3660619 23694687

[4] Geyer LL, Schoepf UJ, Meinel FG, Nance JW Jr, Bastarrika G, Leipsic JA, et al. State of the art: iterative CT reconstruction techniques. Radiology. 2015;276(2):339–57. 10.1148/radiol.2015132766. 26203706

[5] Kataria B, Althén JN, Smedby Ö, Persson A, Sökjer H, Sandborg M, et al. Assessment of image quality in abdominal computed tomography: effect of model-based iterative reconstruction, multi-planar reconstruction and slice thickness on potential dose reduction. Eur J Radiol. 2020;122:108703. 10.1016/j.ejrad.2019.108703. 31810641

[6] Arndt C, Güttler F, Heinrich A, Bürckenmeyer F, Arndt C, Güttler F, et al. Deep learning CT image reconstruction in clinical practice. Fortschr Röntgenstr. 2021;193(03):252–61. 10.1055/a-1248-2556. 33302311

[7] McCleary CM, Chunara MH, Gravell RJ, Rauf A, Cushnie A, Staley C, et al. The future of CT: deep learning reconstruction. Clin Radiol. 2021;76(6):407–15. 10.1016/j.crad.2021.01.010. 33637310

[8] Van Stiphout JA, Driessen J, Koetzier LR, Ruules LB, Willemink MJ, Heemskerk JWT, et al. The effect of deep learning reconstruction on abdominal CT densitometry and image quality: a systematic review and meta-analysis. Eur Radiol. 2022;32(5):2921–29. 10.1007/s00330-021-08438-z. 10.1007/s00330-021-08438-z PMC9038933 34913104

[9] Golbus AE, Steveson C, Schuzer JL, Rollison SF, Worthy TY, Jones AM, et al. Ultra-low dose chest CT with silver filter and deep learning reconstruction significantly reduces radiation dose and retains quantitative information in the investigation and monitoring of lymphangioleiomyomatosis (LAM). Eur Radiol. 2024;34(9):5613–20. 10.1007/s00330-024-10649-z. PMC11364713 38388717

[10] Cirino HJ, Matsumoto JKN, Nomura CH, Scoppetta LR. Impact of silver beam on radiation dose reduction in chest computed tomography: first impressions. J Cancer Prev Curr Res. 2023;14(3):74‒75. 10.15406/jcpcr.2023.14.00524.

[11] Otgonbaatar C, Jeon SH, Cha SJ, Shim H, Kim JW, Ahn JH, et al. Image quality and radiation dose of reduced-dose abdominopelvic computed tomography (CT) with silver filter and deep learning reconstruction. Sci Rep. 2025;15(1):25757. 10.1038/s41598-025-11184-7. PMC12267450 40670595

[12] Szolar DH, Kammerhuber F. Quantitative CT evaluation of adrenal gland masses: a step forward in the differentiation between adenomas and nonadenomas? Radiology. 1997;202(2):517–21. 10.1148/radiology.202.2.9015083. 9015083

[13] Joshi A, Kale S, Chandel S, Pal DK. Likert scale: explored and explained. Br J Appl Sciamp; Technol. 2015;7(4):396–403. 10.9734/BJAST/2015/14975.

[14] Kawamoto K, Sato H, Kogure Y. Usefulness of Ag additional filter on image quality and radiation dose for low- dose chest computed tomography. J Comput Assist Tomogr. 2024;48(2):236–43. 10.1097/RCT.0000000000001538. 37551143

[15] Nomura K, Fujii K, Got T, Tsukagoshi S, Ota H, Iwabuchi Y, et al. Radiation dose reduction for computed tomography localizer radiography using an Ag additional filter. J Comput Assist Tomogr. 2021;45(1):84–92. 10.1097/RCT.0000000000001026. 33475316

[16] Oshima Y, Ohno Y, Takenaka D, Ito Y, Kimata H, Fujii K, et al. Capability for dose reduction while maintaining nodule detection: comparison of silver and copper X-ray spectrum modulation filters for chest CT using a phantom study with different reconstruction methods. Eur J Radiol. 2023;166:110969. 10.1016/j.ejrad.2023.110969. 37454556

[17] Barat M, Cottereau A, Gaujoux S, Tenenbaum F, Sibony M, Bertherat J, et al. Adrenal mass characterization in the Era of quantitative imaging: state of the art. Cancers (Basel). 2022;14(3):569. 10.3390/cancers14030569. PMC8833697 35158836

[18] Johnson PT, Horton KM, Fishman EK. Adrenal Mass imaging with multidetector CT: pathologic conditions, pearls, and pitfalls. RadioGraphics. 2009;29(5):1333–51. 10.1148/rg.295095027. 19755599

[19] Afifi MB, El-Farrashc AH, Abdelrazekc A, Deiab N, El-Hafez AIA. The effects of CT X-ray tube voltage and current variations on the relative electron density (RED) and CT number conversion curves. J Radiat Res Appl Sci. 2020;13(1):1–11. 10.1080/16878507.2019.1693176.

[20] Jaafar AM, Elsayed H, Khalil MM, Yaseen MN, Alshewered A, Ammar H, et al. The influence of different kVs and phantoms on computed tomography number to relative electron density calibration curve for radiotherapy dose calculation. Precis Radiat Oncol. 2022;6(4):289–97. 10.1002/pro6.1177.

